# Mothers with a history of trauma and their children: a systematic review of treatment interventions

**DOI:** 10.3389/fpsyg.2024.1293005

**Published:** 2024-07-16

**Authors:** Elisa van Ee, Eline M. Meuleman

**Affiliations:** ^1^Psychotraumacentrum Zuid Nederland, 's-Hertogenbosch, Netherlands; ^2^Behavioural Science Institute, Radboud University, Nijmegen, Netherlands

**Keywords:** psychotrauma, mother–child relationship, intervention, systematic review, PTSD

## Abstract

**Introduction:**

Mothers with a history of trauma frequently face significant challenges in their relationships with their children. Therefore, it is crucial for trauma-exposed mothers and their young children to receive adequate trauma-informed treatment. This review aimed to examine the effects of trauma-informed interventions on improving the mother–child relationship among mothers with a history of trauma and their young children under 6 years old.

**Methods:**

The study analyzed 15 articles that met the eligibility criteria, encompassing a sample size of 1,321 mothers. The systematic GRADE approach was used to rate the certainty of evidence in this systematic review.

**Results:**

The study found that while some interventions demonstrated small to large effects, the quality of evidence was only moderate. The most promising interventions were Parent–Child Interaction Therapy (PCIT), Child–Parent Psychotherapy (CPP), and Maternal Empowerment Program (MEP), which all included elements of psychotherapy, psychoeducation, and skills training.

**Discussion:**

The study underscores the importance of understanding the needs of mother–child dyads affected by trauma and tailoring interventions to meet those needs. Overall, the literature suggests that interventions combining psychotherapeutic, psychoeducational, and skills-training components are most effective in improving mother and child-related outcomes for mothers with a history of trauma and their young children. The review provides recommendations for future research and emphasizes the importance of considering the mother–child relationship in trauma-informed interventions.

## Introduction

Attachment theory emphasizes the importance of establishing a secure attachment relationship between a parent and a child. A secure parent–child relationship is associated with positive outcomes in various developmental domains, including socio-emotional development and language skills (Bowlby, 1969; Belsky and Fearon, 2002; De Falco et al., 2014). Conversely, an insecure attachment relationship increases the likelihood of the child developing psychopathological symptoms throughout their lifespan (Bosquet Enlow et al., 2011; Kerstis et al., 2018; Luyten et al., 2020). Therefore, it is essential for parents to adopt sensitive and responsive parenting practices to form a secure attachment relationship and maintain a positive and stable parent–child relationship (Ainsworth et al., 1978; Main, 1990; Doinita and Maria, 2015).

Mothers who have experienced trauma, a long term emotional response to a distressing event, are more likely to establish less optimal parent-child relationships with their (young) children (Bosquet Enlow et al., 2013; van Ee et al., 2016). According to the American Psychiatric Association, trauma is defined as a long term emotional response to a distressing event, such as an accident, crime, natural disaster, sexual or physical abuse. Long term reactions include intrusive memories and reexperiencing of the event, avoidance of reminders, negative changes in thinking and mood, and physical or emotional arousal (American Psychiatric Association, 2013). Research shows that women with a history of trauma tend to exhibit caregiving behaviors that foreshadow the development of insecure attachment dynamics with their children (Schechter et al., 2007; Davies et al., 2008). These mothers with a history of trauma tend to engage in insensitive and unresponsive parenting practices, often characterized by more rejecting and avoidant interactions with their offspring (Green and Goldwyn, 2002; Ayers et al., 2006; Schwerdtfeger et al., 2013; van Ee et al., 2016). This decrease in sensitive and responsive caregiving may result from trauma reminders or symptoms linked to the trauma (Kosslyn, 2005; Janssen et al., 2022). Fear induced by intrusive, avoidant symptoms, and trauma-related symptoms, like dissociation, can lead to parenting behaviors that either frighten the child or stem from the mother’s fear, resulting in insecure or even disorganized attachment relationships (Main and Hesse, 1990; Madigan et al., 2006; Bosquet Enlow et al., 2013). Such frightened and frightening behavior may represent an indirect pathway through which past traumatic events influence mother–child relationships (Bosquet Enlow et al., 2013; Samuelson et al., 2016).

Even though previous research has predominantly focused on the connections between maternal traumatic experiences in close relationships, adverse associations between traumatic experiences and mother–child interactions have emerged across various types of trauma. Associations between traumatic experiences such as child maltreatment or adult interpersonal violence (IPV) and parenting outcomes (Wilson et al., 2017) align with attachment theory. This theory posits that exposure to interpersonal traumas significantly influences a mother’s parenting behavior. Studies on other populations, such as veterans and refugees, further extend these findings. They reveal associations between posttraumatic stress symptoms stemming from warfare-related experiences and insensitive, unstructured, and hostile interactions between parents and children (van Ee et al., 2012, 2018).

Attachment holds particular significance as a developmental milestone in early childhood. Researchers have emphasized that maternal attachment is vital during this formative period, as experiences during these early years can exert lasting effects on a child’s mental and physical well-being (Belsky and Fearon, 2002; Bosquet Enlow et al., 2011, 2013). Therefore, research underscores the imperative nature of early interventions aimed at improving the mother–child relationship for mothers with a history of trauma (Ghosh Ippen et al., 2011; van Ee et al., 2012; Cross et al., 2018; Overbeek et al., 2019). Such interventions may mitigate challenges arising from experiencing trauma, especially among toddlers and preschoolers (Prior et al., 1992; Bosquet Enlow et al., 2011, 2013).

While research on trauma-informed interventions for mothers is expanding, there remains a dearth of research on interventions explicitly designed for the mother–child relationship for mothers with young children (0–6 years old). Trauma-informed interventions reflect knowledge of trauma and its effect on survivors in sensitive ways of understanding and engaging with the mother (Classen and Clark, 2017). A comprehensive overview of such interventions could provide valuable insights for clinical practice. In conclusion, early trauma-informed interventions that focus on improving the mother–child relationship (on a substantive level) are crucial for mothers with a history of trauma and their young children, and as of now, no review has synthesized these critical interventions. In all, this review aims to paint an overarching picture of trauma-informed interventions designed for the mother–child relationship. The objective of the study is to answer the following question: Is there empirical support suggesting that interventions aimed at enhancing the relationship between mothers with a history of trauma and their child(ren) under the age of six result in positive post-intervention outcomes for both the mother *and* the child?

## Methods

### Eligibility criteria

The methodology adopted in conducting this systematic review was following the PRISMA (Preferred Reporting Items for Systematic Reviews and Meta-Analyses) guidelines (Prior et al., 1992). Inclusion criteria for articles were: (1) testing of intervention effectiveness; (2) the content and sessions of the intervention (i.e., the intervention manual) explicitly aimed to enhance the mother–child relationship; (3) the intervention was trauma-informed, it took the trauma history, trauma response, and ability to engage in treatment into account, and targeted toward mothers with a history of trauma (and not their children); (4) the mean age of children included was less than 6 years old; (5) assessment of outcomes for both the mother and/or child was performed; (6) utilization of quantitative, qualitative, or mixed methodologies; and (7) the intervention manual and article were written in English. To ensure the potential for international clinical implementation, only English language interventions were included, while case studies were excluded from the analysis.

Regarding the interventions, the search strategy adopted in this review was inclusive, encompassing all interventions aimed at improving parenting behavior and attachment. While all interventions included elements aimed at improving the mother–child relationship, the relationship itself was not necessarily an outcome variable.

### Search strategy

Publicly accessible papers were used as a basis for this systematic review. The databases PsycINFO, Medline, WoS, PTSDpubs-PILOTS, PubMed, and Embase were screened. We conducted searches using the following search string: [(“mother” OR “mothers” OR “motherhood” OR “mother–child” OR “mother–child relationship” OR “attachment” OR “bonding”) AND (“‘posttraumatic stress disorder” OR “PTSD” OR posttraumatic stress symptoms” OR “psychotrauma”) AND (“intervention” OR “interventions” OR “systemic” OR “therapy” OR “therapies” OR “therapeutic”)]. In accordance with the specified inclusion criteria, data was gathered for all outcome domains without any pre-defined limitations. Furthermore, search terms included ‘posttraumatic stress symptoms’ as well as ‘psychotrauma’. The main focus of an intervention should be to enhance the mother–child relationship of mothers who have experienced a traumatic event and, as a consequence, develop posttraumatic stress-related symptomology (not necessarily a diagnosis). A broad combination of search terms was used to ensure all relevant interventions were included, thereby increasing the sensitivity of the research.

Initial searches were completed in January 2022. Moreover, reference lists of included full-text articles were searched and relevant papers were screened for eligibility. Two coders screened each record (the first author, educational level: B.Sc., and a colleague, educational level: M.Sc.). Disagreements on the inclusion of a full-text article were discussed with all authors. The consensus of all involved researchers (three in total, including one senior researcher who is a full professor) was reached regarding the final inclusion of all articles.

### Data analyses

The systematic search process consisted of three phases. Firstly, articles underwent screening based on their titles and abstracts, with those failing to meet the predetermined inclusion criteria being excluded. Subsequently, in the second phase, full-text screenings were performed on all articles that met the inclusion criteria, as well as on any articles where it was unclear whether the inclusion criteria were satisfied. Relevant articles were then incorporated, and their respective reference lists were reviewed to identify additional pertinent sources. Ultimately, one of the authors independently evaluated the included articles using the Grades of Recommendation, Assessment, Development, and Evaluation (GRADE) approach. The outcomes of this evaluation were deliberated with the other author, leading to a final consensus being reached.

The GRADE approach provides a structured, step-wise process to determine the quality of evidence present in an included article (Brożek et al., 2009). The quality of evidence was categorized as high (4), moderate (3), low (2), or very low (1). The article was initially evaluated based on its experimental design, with randomized controlled trials (RCTs) being ranked as high (4) and trials without a control group ranked as low (2). The study could then be downgraded based on its limitations, with one level of downgrade for one serious limitation and two levels for multiple limitations. For example, serious limitations such as data from a single RCT with no randomization description and high attrition rates would result in a downgrade from high to low (two levels). A lack of blinding or a high risk of bias due to the exclusion of confounding variables would result in a single-level downgrade. Other factors, such as a large magnitude of effect and a dose–response gradient, increased the quality of evidence. The quality of evidence for downgraded or upgraded randomized trials and for observational studies is considered moderate (3) and for triple-downgraded randomized trials or downgraded observational studies very low.

For the included articles, a summary of the intervention description and reported outcomes was conducted. Wherever possible, effect sizes were noted or calculated manually. The following rules of thumb were applied: Cohen’s *d* of 0.01 was considered very small, 0.2 was considered small, 0.5 was considered medium, and 0.8 was considered large (Sawilowsky, 2009; Cohen, 2013). For *r*^2^, *R*^2^, and *η^2^*, 0.01 was considered small, 0.06 was considered medium, and greater than 0.14 was considered large (Cohen, 2013). A meta-analysis was not performed due to the diverse outcomes reported and limited interventions designed.

## Results

### Study selection

**Figure 1:**
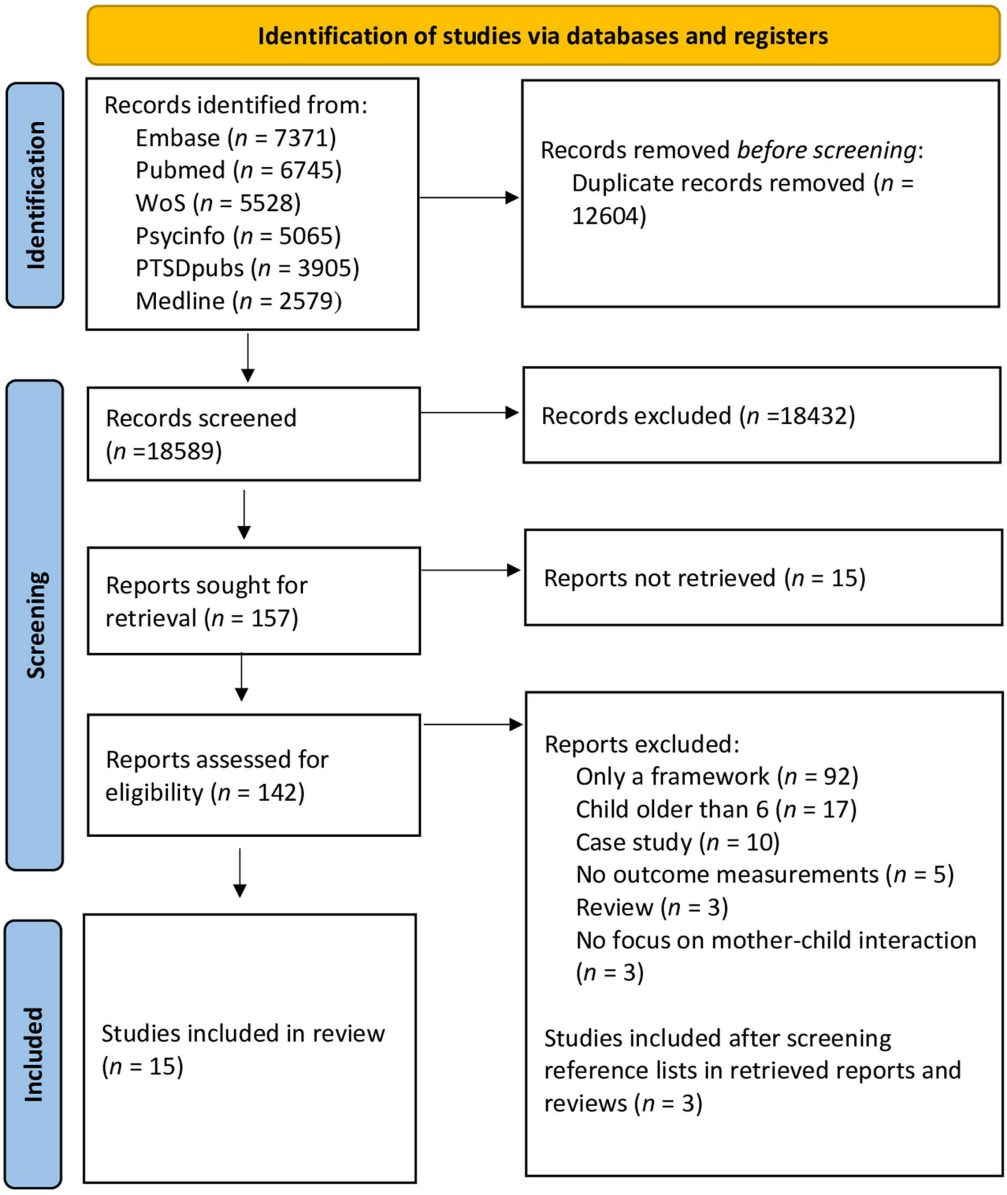
Flow diagram (Page et al., 2021).

The initial search contained a total of 31,193 articles. After removing duplicates, 18,589 articles remained. During the initial screening of titles and abstracts, articles that did not meet the inclusion criteria were removed, with the majority of them lacking a measurement of the intervention’s efficacy. Afterwards, 142 articles remained which were screened in full text. After eliminating articles that did not meet the inclusion criteria, fifteen articles were included in the final review (Figure 1). In this stage, most articles were excluded because they did not empirically evaluate the intervention’s effectiveness.

### Study characteristics

This review identified seven randomized controlled trials (RCTs), seven non-randomized interventions without a control group, and one quasi-experimental intervention. For a full overview of all study characteristics, see Table 1. The interventions span 20 years, from 2001 to 2021. In total, 15 articles were included in the review, covering a sample size of 1,321 mothers. The average sample size per article consisted of 88 mothers. Participants (mothers) were recruited from the United States (*n* = 781), Israel (*n* = 161), Canada (*n* = 120), Bosnia (*n* = 87), Spain or the United States (*n* = 105), and Canada or the United States (*n* = 67). The included mothers had experienced various trauma types: IPV (10 articles), exposure to political violence or war activities (three articles), exposure to at least four traumatic events (one article), and premature birth and hospitalization (one article).

**Table 1:** Included studies.

Author (year)	Intervention	Location	Sample size	Maternal trauma	Sessions	Control group	Assessment points
Cohen et al. (2013)	NAMAL	Israel	70	War-related trauma	Two years, 10 sessions (*Combined intervention*)		Post-test, 1 year follow-up
Cohen and Shulman (2017)	NAMAL	Israel	54	Political violence	Two years, 10 sessions (*Combined intervention*)		Pre- and post-intervention
Dybdahl (2001)	Psychosocial intervention	Bosnia	87	War-related trauma	Five months, weekly sessions (*Separate intervention*)	Medical care only	Pre- and post-intervention
Galano et al. (2021)	MEP	USA + Canada	67	IPV	Five weeks, two sessions from 120 min per week (*Separate intervention*)	Waitlist control	Follow-up measure after 6–8 years
Ghosh Ippen et al. (2011)	CPP	USA	75	At least 4 TSEs	50 weeks, one session/week (*Combined intervention*)	Case management	Pre- and post-intervention, follow-up
Hagan et al. (2017)	CPP	USA	199	IPV	Approximately 36 weeks, 60 min/week (*Combined intervention*)		Pre- and post-intervention
Howell et al. (2014)	MEP	Canada	120	IPV	5 weeks, two sessions from 120 min per week (*Separate intervention*)		Pre- and post-intervention
Lieberman et al. (2005)	CPP	USA	75	IPV	50 weeks, 60 min/week (*Combined intervention*)	Usual care	Pre-intervention, 6 months later
Lieberman et al. (2006)	CPP	USA	50	IPV	50 weeks, 60 min/week. (*Combined intervention*)	Monthly telephone calls	6-month follow-up
Muzik et al. (2015)	Mom Power	USA	99	IPV	10 weeks, 180 min/week +3 individual sessions (*Combined intervention*)		Pre- and post-intervention
Rosenblum et al. (2017)	Mom power	USA	122	IPV	10 weeks, one 3-h session/week + three individual sessions (*Combined intervention*)	Weekly mailings	Pre- and post-intervention
Schechter et al. (2006)	CAVES	USA	32	Violence	Three visits in 1.5 months (*Combined intervention*)		Pre-, during-and post-intervention
Shaw et al. (2013)	Prevention of Trauma	USA and Spain	105	Premature birth and hospitalization	3–4 weeks with one or two 45-to 55-min sessions weekly (*Separate intervention*)	Usual care	Pre- and post-intervention
Timmer et al. (2010)	PCIT	USA	129	IPV	14 to 20 weeks parent–child intervention (*Joint intervention*)	No IPV exposure	Pre- and post-intervention
Waldman-Levi and Weintraub (2014)	FI-OP	Israel	37	IPV	8 weeks, 30 min/week. (*Joint intervention*)	Free play time	Pre- and post-intervention

Sample size is the number of mothers who participated.

### Risk of bias in studies

On average, the 15 articles scored a 3 (‘moderate’) according to the GRADE criteria. See Table 2 for an overview of these studies’ GRADEs, strengths, and limitations.

**Table 2:** Assessment of articles according to GRADE-approach.

Author (year)	Study design	Strengths	Limitations	GRADE
Cohen et al. (2013)	Non-randomized intervention	Manual, 1 year follow-up, multimethod	No control group, convenience sample, no limitation section, self-reported outcomes	2
Cohen and Shulman (2017)	Non-randomized intervention	Manual, continuous measure exposure to trauma, confounding variables	No control group, no limitation section, self-reported outcomes,	2.5
Dybdahl (2001)	RCT	Randomized, multimethod, baseline differences, blind testers	Contamination of effects, maternal outcomes only through interviews, effect sizes could not be calculated	2.5
Galano et al. (2021)	RCT	Manual, randomized, 8-year-follow up, baseline differences	Convenience sample, self-reported outcomes, insufficient power, attrition over time	3
Ghosh Ippen et al. (2011)	RCT	Manual, randomized, treatment fidelity	Small sample size, self-reported outcomes, limited follow-up analyses, dichotomization of children	3
Hagan et al. (2017)	Non-randomized intervention	Manual, treatment fidelity, large sample, addresses measurement error and missing data	No control group, self-reported outcomes	3
Howell et al. (2014)	Non-randomized intervention	Manual, comparison group, outcome measures for parenting, confounding variables	No control group, convenience sample, self-reported outcomes, low generalizability, sample retention	2.5
Lieberman et al. (2005)	RCT	Manual, randomized, treatment fidelity, community sample	Variable number of therapy sessions, self-reported outcomes	4
Lieberman et al. (2006)	RCT, follow-up	Manual, randomized, treatment fidelity, follow-up measures, community sample	Variable number of sessions, no new interviews, groups differed on group sex and age of children, self-reported outcomes	3.5
Muzik et al. (2015)	Non-randomized intervention	Manual, high retention, feasibility study	No objectively coded mother–child interactions, small sample, no control group	2.5
Rosenblum et al. (2017)	RCT	Manual, randomized, community sample, attendees not differ from drop-outs	High attrition for post-assessment, no child outcomes, no follow-up assessment	4
Schechter et al. (2006)	Non-randomized intervention	Interrater reliability, community sample	No control group, only one outcome	2
Shaw et al. (2013)	RCT	Manual, randomized	Active comparison group, self-reported outcomes, short-term data, ethnic groups underrepresented	4
Timmer et al. (2010)	Quasi-experimental	Identified barriers to intervention completion, equal groups, no dosage effect	No randomization, no information about severity or duration of violence, no follow-up data, self-reported outcomes	3.5
Waldman-Levi and Weintraub (2014)	Non-randomized intervention	Fidelity of treatment, playroom program as control, observational measure of children’s play behavior	No control group, convenience non-randomized sampling, no follow-up data	2.5

### Interventions’ description and results

#### Outcomes overall

The intervention effects were analyzed on various outcomes. Some interventions resulted in either reduced maternal posttraumatic stress symptoms (*n* = 5; CPP, Psychosocial Intervention, MEP, Prevention of Trauma-Intervention, Mom Power) or improved maternal parenting practices (*n* = 4; MEP, FI-OP, Mom Power, NAMAL, CAVES). Three interventions (CPP, Prevention of Trauma-Intervention, and Mom Power) had an impact on maternal depression symptoms, while one intervention (PCIT) had an impact on maternal distress. In terms of child-related outcomes, some interventions showed a decrease in behavioral problems (*n* = 4; CPP, Psychosocial Intervention, PCIT, NAMAL). One intervention (CPP) was effective in reducing posttraumatic stress symptoms, depression symptoms, and/or co-occurring diagnoses in children, and another intervention (FI-OP) improved children’s play behavior.

#### NAMAL

NAMAL is an acronym derived from the Hebrew phrase “Let us Create Space for Play.” Its primary objective is to cultivate emotional openness and encourage playfulness between mothers and their children. This is achieved through several key components, including the co-creation of a shared narrative around trauma, providing psychoeducation on typical trauma responses in young children, and equipping mothers with skills for attuning to and reflecting on emotions, as well as enhancing coping and stress management abilities (Cohen et al., 2013; Cohen and Shulman, 2017).

Two studies have been conducted to assess the effectiveness of NAMAL. The first study involved 70 mothers and their children who had been exposed to recurrent rocket attacks (Cohen et al., 2013), while the second study involved 54 mothers and their children exposed to political violence (Cohen and Shulman, 2017). Both studies utilized a non-randomized intervention design. Qualitative results indicated that children participating in NAMAL displayed a more positive mood, increased expressions of excitement, reduced temper tantrums, and an improved ability to listen (Cohen et al., 2013). Quantitative results showed that mothers demonstrated a significant improvement in emotional availability (medium effect, *d* = 0.36) and their children experienced fewer behavioral problems (large effect, *d* = 0.93) post-intervention (Cohen and Shulman, 2017). The effect sizes were derived through a manual calculation of Cohen’s *d* for two-tailed *t*-tests, given that the studies did not provide any effect size measurements.

#### Psychosocial intervention

The second intervention is the psychosocial intervention described by Dybdahl (2001). Group leaders in the psychosocial intervention provide mothers with information about common posttraumatic needs and problems of their children during semi-structured group meetings. Discussions focus on various topics, including the mothers’ mental health, mother–child interaction, and coping strategies. Furthermore, mothers are taught communication and interaction skills.

The hyperarousal trauma symptoms of mothers (*N* = 87, all experienced severe war activities) decreased after taking part in the non-randomized intervention (Dybdahl, 2001). There were no effect sizes mentioned to interpret this decline and these could not be calculated since the *n* of each group was not reported. Interviewers also reported that children had fewer behavior problems.

#### Moms’ empowerment program (MEP)

Within MEP, mothers openly discuss their parenting skills and the impact of IPV on their child’s development in a group setting which promotes the development of new parenting practices and a supportive parenting environment (Howell et al., 2014). In addition, mothers receive information about the typical development of children, stress reduction, and problem-solving of parenting issues (Galano et al., 2021). Also, mothers acquire coping skills that contribute to their stress management during difficult parent–child interactions.

The MEP was evaluated in two articles in which, respectively, 120 and 67 mothers participated who experienced interpersonal trauma (Howell et al., 2014; Galano et al., 2021). The first showed that MEP induced a significant and positive change in maternal self-reported parenting practices, such as involvement, discipline, and positive parenting (*d* = 0.14, indicating a small effect; 38). The second article focused on intervention effects 8 years after concluding the intervention. Results showed that only those mothers who had attended more than eight sessions had fewer reexperiencing and hyperarousal symptoms. Both effect sizes were medium (*R*^2^ = 0.07; 38). There was no general intervention effect.

#### Child–parent psychotherapy (CPP)

The CPP sessions are directed by parent–child interactions and consist of individual appointments with the mother, in which clinicians inform mothers about assessment findings and treatment plans, and joint child–parent sessions (Lieberman et al., 2005). During these joint sessions, clinicians attempt to alter maladaptive (dyadic) behaviors and create a joint trauma narrative.

Four articles described and evaluated CPP, with sample sizes ranging from 50 to 199 dyads (Lieberman et al., 2005, 2006; Ghosh Ippen et al., 2011; Hagan et al., 2017). Results of an RCT showed that CPP induced changes for both the mother (who experienced at least four traumatic events) and the child. Mothers who completed CPP had a lower chance of having posttraumatic stress (*η^2^* = 0.31) or depression symptoms (*η^2^* = 0.24) afterward. Children also had fewer posttraumatic stress symptoms (*η^2^* = 0.22), depression symptoms (*η^2^* = 0.08), and co-occurring diagnoses after completing the intervention (*η^2^* = 0.15; 26). After 6 months, there was a decline in behavior problems (*η^2^* = 0.25). These effects were all large, except for the child’s depression symptoms. Results of Hagan et al. (2017), which were based on a latent variable approach with a good model fit, demonstrated that CPP led to a decrease in children’s PTSS re-experiencing, hyperarousal, and avoidance symptoms, and a reduction in maternal PTSD. The articles by Lieberman et al. (2005, 2006), which described an RCT (75 dyads) and its follow-up (50 dyads), showed that CPP led to a decrease in children’s trauma symptoms (*d* = 0.63) and behavior problems (*d* = 0.41 and *d* = 0.24), and a reduction in maternal avoidant symptoms (*d* = 0.5) and global symptom severity (*d* = 0.38). The effect sizes of these found results ranged from small to medium.

#### Mom power

Mom Power aims to promote a secure attachment between mother and child (Muzik et al., 2015). Together with facilitators, mothers identify past experiences that could affect their children. In group sessions, mothers reflect on their behavior and feelings and gain insight into their mother–child relationship. Besides these therapeutic parts of the intervention, facilitators also present educational material focused on how to be a secure base and haven for a child and how to create a warm bond. Moreover, facilitators teach mothers about self-care skills such as mindfulness, guided breathing, and affect regulation (Rosenblum et al., 2017).

Muzik et al. (2015) and Rosenblum et al. (2017) evaluated the intervention ‘Mom Power,’ in which, respectively, 99 and 122 mothers took part, who had all experienced IPV. The article of Rosenblum et al. (2017) was an RCT; whereas, Muzik et al. (2015) described the outcomes of a non-randomized intervention. Accordingly, Mom Power led to a decrease in posttraumatic stress symptoms (*d* = 0.27), depression symptoms (*d* = 0.34), maternal self-rated caregiver helplessness (*d* = 0.22), and interviewer-rated caregiver helplessness (*d* = 0.32; 44). No effect sizes were mentioned in the article, the reported sizes are thus manually calculated and all reflect small effects. In the second analysis (Rosenblum et al., 2017), Mom Power led to a decline in maternal PTSD symptoms in the Mom Power condition only. The effect was small (*d* = 0.20).

#### Clinician assisted videofeedback exposure session (CAVES)

The clinician assisted videofeedback exposure sessions (CAVES) -intervention utilizes video feedback to alter maternal attributions. These maternal attributions reflect perceptions of the child’s behavior and can be distorted by a mother’s own relational experiences (Schechter et al., 2006). In CAVES, mothers are, for example, exposed to their child’s reactions to separation. This reaction might trigger posttraumatic stress, and therefore the clinician will model and stimulate reflective functioning. In addition, mothers and clinicians reflect on a moment of sub-optimal play.

The 32 mothers who participated in the non-randomized intervention of CAVES had fewer negative maternal attributions after the intervention (Schechter et al., 2006). Based on the reported *F*-values and a confidence interval of 90%, we calculated the *η^2^* which equals 0.23, indicating a large effect.

#### Prevention of trauma

The six-session treatment developed by Shaw et al. (2013) combines principles of trauma-focused CBT with psychoeducation. The sessions focus on (1) psychoeducation to instruct mothers about posttraumatic stress and feelings and thoughts related to their infant, (2) cognitive restructuring aimed at changing maladaptive cognitions, (3) muscle relaxation techniques, and (4) the development of a mother’s trauma narrative. The intervention’s main goal is to enhance the mother-infant relationship by altering the mother’s negative perceptions.

Mothers (*N* = 105; who had the traumatic experience of premature birth and hospitalization) who participated in the Prevention of Trauma-intervention (RCT) of Shaw et al. (2013) had less trauma (*d* = 0.41) and depression symptoms (*d* = 0.59) post-intervention. Both effects had a medium effect size. Mothers with higher ratings of baseline parental stress benefited more from the intervention compared to mothers with lower ratings.

#### Parent–child interaction therapy (PCIT)

In PCIT, a therapist coaches mothers and children to change their dysfunctional relationships (Timmer et al., 2010). The first part of PCIT aims to enhance the parent–child relationship, whereas the second part attempts to increase child compliance. Both parts start with didactic training and coaching during play. Mothers practice communication skills, such as reflections and praises, and learn how to manage their children’s behavior.

After completing the PCIT intervention, in which 129 mothers who experienced IPV and their children participated, mothers reported they had less psychological distress (*η^2^* = 0.16), and their children had significantly fewer behavioral problems (*η^2^* = 0.29; 48). Both effect sizes were large and based on a quasi-experimental pre-post design.

#### Family intervention for improving occupational performance (FI-OP)

The FI-OP is designed to improve children’s play skills and playfulness behavior, by letting the child and mother engage in joint play (Waldman-Levi and Weintraub, 2014). Mother–child interaction, reciprocity, playfulness, and play skills are all addressed during play, by using mediation, modeling, consultation, environmental adaption, reframing, enabling, and reflection. Moreover, mothers receive psychoeducation about the importance of play and interaction. Lastly, mothers acquire skills on how to provide praise for appropriate behavior.

Results of a non-randomized study demonstrated that FI-OP promoted children’s play skills in the symbolic dimension and that the interaction between mother and child significantly improved after the dyad’s participation (Waldman-Levi and Weintraub, 2014). After participating in FI-OP, mothers were more sensitive and better able to set limits during mother–child interaction. We calculated Cohen’s *d* for the difference in scores between the intervention and control group for sensitivity and limit setting and these were, respectively, 0.75 and 0.54 (medium effects). This article included 37 mothers who experienced IPV and their children.

## Discussion

Prior research has consistently shown that mothers with a history of trauma face challenges in their relationships with their children (van Ee et al., 2012; Samuelson et al., 2016; Wilson et al., 2017). Various authors have emphasized the importance of trauma-informed interventions that prioritize improving the mother–child relationship, especially during early childhood (Ghosh Ippen et al., 2011; Cross et al., 2018; Overbeek et al., 2019). Despite this recognition, there is a scarcity of interventions specifically designed to enhance the mother–child relationship for these mothers and their young children (0–6 years old).

### Main findings

In this systematic review, nine trauma-informed interventions were identified that aim to strengthen the mother–child relationship for mothers who have experienced trauma alongside their young children. Due to the limited body of literature in this area, drawing definitive conclusions about these interventions is challenging. The effect sizes of the interventions varied: one intervention (Mom Power) yielded small effects, while others exhibited small and medium effects (CPP, MEP), medium effects (Prevention of Trauma, FI-OP), medium and large effects (NAMAL), or exclusively large effects (CAVES, PCIT). The effect size for the Psychosocial intervention was not reported and therefore could not be calculated. Additionally, the GRADE approach was employed to assess the interventions, with the quality of evidence consistently indicating relatively low levels (≤2.5) for the interventions Prevention of Trauma, NAMAL, CAVES, and FI-OP. While CPP and Mom Power each received one low-GRADE evaluation, they were further supported by studies with higher-quality evidence. Consequently, based on the GRADE approach, there is only a weak recommendation for the use of NAMAL, CAVES, and FI-OP for mothers with a history of trauma and their children.

Considering both effect sizes and GRADE evaluations, PCIT, CPP, and MEP appear to be the most promising interventions for enhancing mother and child-related outcomes in mothers with a history of trauma and their young children. These trauma-informed interventions seem to result in positive post-intervention outcomes for both the mother and the child, however, due to the limited evidence available, a shared decision-making process is vital before selecting an intervention. These three interventions share common elements of psychotherapy, psychoeducation, and skills training. Psychoeducation and skills training encompass aspects such as social support, play skills, coping skills, and relaxation techniques, while psychotherapeutic interventions place a strong emphasis on the therapeutic relationship. These interventions offer mothers a corrective emotional experience by providing a secure base and addressing cognitive restructuring and reflective functioning (Muzik et al., 2015; Norcross and Lambert, 2019; van Ee and Blokland, 2019). Therapists serve as models for addressing the child’s needs by attending to the mother’s needs, focusing on sensitive parenting, and addressing maternal mental health. Consequently, these interventions have the potential to positively impact the functioning of the mother, child, and family system (Smith et al., 2010).

The dearth of interventions aimed to strengthen the mother–child relationship may be attributed to an individualistic approach to trauma experiences and their consequences, which tends to overshadow a more comprehensive, social-ecological perspective on the repercussions of trauma experiences (Smith et al., 2010; Maercker and Hecker, 2016; Shallcross et al., 2016; Wang et al., 2021). Most posttraumatic stress disorder (PTSD) treatment protocols predominantly focus on individual experiences and associated symptoms, often overlooking the systemic impact of traumatic events. Consequently, it is crucial to intensify efforts to comprehend the needs of these mother–child dyads, as these needs should guide the objectives of interventions. In summary, despite consistent research findings indicating difficulties in mother–child relationships among mothers with a history of trauma, systemic knowledge about the needs of these dyads and the most effective means of addressing these needs remains limited. The existing body of literature, although modest, suggests that the most promising interventions are those that incorporate a combination of psychotherapeutic, psychoeducational, and skills-training components.

### Future research and limitations

Further research is imperative in several domains. Firstly, there is a need for a deeper understanding of the mechanisms underpinning the strained mother–child relationship in mothers with a history of trauma and how these mechanisms can inform the development of effective interventions. For instance, the role of mentalization (the process of understanding mental states in oneself and others; Zeegers et al., 2017) in shaping secure attachment relationships between mothers and children should be explored. Schechter and Willheim (2009) have argued that enhancing the mentalization capacity of trauma-exposed is necessary to improve mother–child attachment relationships. Moreover, future studies should disentangle the effects of interventions on reducing posttraumatic stress-related symptomatology and their direct impact on the mother–child relationship or parenting behavior. It remains unclear whether treating PTSD in isolation is sufficient or if it should be complemented with mother–child sessions to enhance the attachment bond. Additionally, to comprehensively assess the impact of interventions on the mother–child relationship, standardized assessments of that relationship should be included as outcome measures. Although many interventions focus on the mother–child relationship, it is often not measured directly, making it challenging to determine the effectiveness of the intervention. In conclusion, further research is essential to gain deeper insights into the risks in the mother–child relationship, the mechanisms of change, and the effects of various intervention components, in order to provide well-informed recommendations for mothers with a history of trauma and their children.

The present review has several limitations. The sample size of the reviewed articles was small (*M* = 88.1, *SD* = 43.1), thereby limiting the power of the included studies. Additionally, the majority of the articles (9 out of 15) focused on participants who had experienced intimate partner violence, which may restrict the generalizability of the results to other populations. The assessment of PTSD was not standardized across the articles, as none of them employed a specific PTSD cutoff score for participant selection. Further exploration of the traumatic experiences and PTSD symptoms of mothers is warranted, as those with higher symptom severity may have different needs compared to those with milder symptoms. Furthermore, the absence of control groups in seven studies raises concerns about the reliability of the results. Finally, the findings primarily relied on self-report measures, which may not provide an entirely accurate reflection of the mother–child relationship. Therefore, it is crucial to incorporate more objective and direct assessments, especially of the mother–child relationship, to accurately evaluate the impact of interventions aimed at enhancing this relationship.

Future research should address these methodological limitations by utilizing standardized and comparable measurements, employing experimental designs with control groups, and examining the functioning of both the mother and child, as well as their relationship. To enhance data validity and reliability, a multimethod approach that combines various measurement techniques, such as self-reports along with observations of a 5-min mother–child interaction during shared picture-book reading (Rasheed and Yousafzai, 2015), is recommended. Additionally, other relevant factors like mentalization and the child’s attachment should be considered. Furthermore, it is advisable to report effect sizes to better understand the magnitude of differences associated with the findings. Finally, further research is necessary to identify the essential components of interventions for optimal outcomes in mothers with specific traumatic experiences.

## Author contributions

EE: Writing – original draft, Writing – review & editing. EM: Writing – original draft, Writing – review & editing.
